# Longitudinal association between motor and obsessive compulsive symptoms in patients with psychosis and their unaffected siblings

**DOI:** 10.1007/s00406-018-0898-y

**Published:** 2018-05-29

**Authors:** Marije Swets, Frederike Schirmbeck, Jack Dekker, Lieuwe de Haan, Genetic Risk and Outcome of Psychosis (GROUP) Investigators, René S. Kahn, Jim van Os, Richard Bruggeman, Wiepke Cahn, Agna A. Bartels-Velthuis, Inez Myin-Germeys

**Affiliations:** 1 Department of Psychiatry, Academic Medical Centre University of Amsterdam, Amsterdam, The Netherlands; 2 Arkin Institute for Mental Health, Amsterdam, The Netherlands

**Keywords:** Psychotic disorders, Schizophrenia, Obsessive compulsive disorder, Obsessive compulsive symptoms, Motor symptoms, Akathisia

## Abstract

Little is known about the co-prevalence of obsessive compulsive symptoms (OCS) and motor symptoms in patients with psychotic disorders. Cross-sectional associations between OCS and motor symptoms were assessed at baseline and at 3 years follow-up in patients (*n* = 726) with psychotic disorders and in their unaffected siblings (*n* = 761) from the Dutch Genetic Risk and Outcome of Psychosis (GROUP) study. Furthermore, longitudinal associations between changes in OCS and motor symptoms were evaluated. At baseline, OCS was not associated with any motor symptom (akathisia, dyskinesia, parkinsonism or dystonia) in patients. At follow-up, patients with OCS reported significantly more akathisia. Dividing the patients into four groups—no OCS, OCS remission with OCS only at baseline, OCS de novo with OCS only at follow-up and a persistent OCS group—revealed that the OCS de novo group already reported more akathisia at baseline compared to the no-OCS group. At follow-up, both the OCS de novo and the persistent OCS group reported more akathisia. These results remained significant after correcting for relevant confounders clozapine, GAF score, PANSS-negative score and IQ. Motor symptoms at baseline were significantly associated with OCS at follow-up, but not the other way around. In siblings, OCS at baseline was associated with akathisia, but this association was lost at follow-up. Results suggest that motor symptoms might precede co-occurring OCS in patients with psychotic disorders. However, no inference can be made about causality, and further prospective research is needed to investigate this assumption.

## Introduction

Since the introduction of the DSM-III-R in the 1990s which allowed for comorbid diagnosis with schizophrenia, there has been an increasing research interest in the combination of psychosis and obsessive compulsive symptoms (OCS). Comorbid obsessive compulsive disorder (OCD) in schizophrenia is present in about 13.5% of cases [1, 43], roughly six times more than in the general population [20]. Conversely, in OCD the increased risk of developing schizophrenia is 6.9 [26]. Curiosity regarding the nature and possible implications of the co-existence of the two disorders has led researchers to explore potentially related characteristics. A strong and persistent association between OCS and specific other characteristics in patients and their siblings would support the hypothesis that the combination of schizophrenia and OCS forms a distinct subtype [7]. Like neurocognition [37] or severity of psychotic symptoms [10], one of the characteristics of interest in research concerning OCS comorbidity in psychosis concerns motor symptoms.

The first description of motor symptoms, specifically parkinsonism, akathisia, dyskinesia, catatonia and dystonia in schizophrenia dates back to over a century ago. Parkinsonism is a condition of tremor, motor rigidity and poverty of motion. Akathisia is a feeling of restlessness and an urgent need to move. Dyskinesia is a condition of involuntary repetitive dyskinetic movements. Catatonia is a state of immobility, and dystonia consists of sustained or repetitive muscle contractions. First-generation anti-psychotic drugs, specifically, are associated with motor side-effects. However, in the mid-1980s a group of 47 anti-psychotic naïve chronic patients was studied. Their prevalence of tardive dyskinesia was 51%, only slightly less than matched patients on anti-psychotics [47]. Ever since, motor symptoms are regarded as being commonly present during the course of schizophrenia, and only partially linked to anti-psychotic use.

OCD is known to be associated with primary movement disorders, manifesting tics and chorea. Tourette’s syndrome is the most prominent example [23]; others are Sydenham’s chorea [42], Huntington’s disease [2] and potentially Parkinson’s disease [24]. Unlike schizophrenia though, “plain” OCD is not specifically associated with motor symptoms apart from the above-mentioned motor syndromes and tics. In short, both schizophrenia and OCD are associated with motor symptoms, but the type of motor symptoms differs. OCD is associated with tics/Tourette’s syndrome and Huntington’s disease, but in patients with schizophrenia other motor symptoms are more characteristic, such as stereotypies, catatonia and extrapyramidal signs. As in OCD, the inhibitory system is involved in tics. In schizophrenia, the motor involvement and related lesions are more extensive. Dysregulation of dopaminergic neurotransmission in the basal ganglia is thought to be involved in schizophrenia, OCD and motor symptoms. Abnormalities in the orbitofrontal cortex [36] and the cortico-striato-thalamic-cortical circuitry are relatively OCD specific, while in schizophrenia the neurobiological system involved concerns the frontal cortex, basal ganglia, thalamus and cerebellum, the dorsolateral prefrontal cortex, (pre-)motor cortex and the connecting white matter tracts [12, 45]. A link between schizophrenia and comorbid OCS and motor symptoms is logical because both disorders are associated with motor symptoms.

To the best of our knowledge, eight studies have addressed motor symptoms in schizophrenia patients with OCS [22, 27, 28, 30, 33, 34, 39, 44] (Table 1). The results of these studies appear to be inconclusive; with regard to parkinsonism and dyskinesia, about half the studies report significant higher prevalence’s in patients with comorbid OCS. Concerning dystonia [44] and catatonia [22], only one study reported results.

**Table 1:** Previous findings concerning the association between OCS and motor symptoms in patients with psychotic disorders

First author; publication year; no. of patients	Dyskinesia	Akathisia	Dystonia	Parkinsonism	Catatonia
	Mean AIMS (SD)	Mean BAS (SD)	Mean SARS (SD)		
Krüger 2000, *n* = 76	+ (AIMS)	+ (HAS)	NA	+ (SARS)	+ (CRS)
− OCS	0.5 (0.8)			4.4 (4.0)	
+ OCS	1.6 (1.2)			7.5 (8.8)	
Mukhopadaya 2009, *n* = 59	+ (AIMS)	NA	NA	+ (SARS)	NA
− OCS	1.3 (2.9)			2.6 (2.6)	
+ OCS	4.4 (4.4)			5.1 (3.3)	
Ohta 2003, *n* = 61	+ (AIMS)	− (BAS)	NA	+ (SARS)	NA
− OCS	1.7 (2.8)	0.3 (0.9)		2.5 (3.0)	
+ OCS	6.2 (8.1)	0.2 (0.6)		4.3 (4.8)	
Patel 2010, *n* = 22	+ (AIMS) p = 0.06	NA	NA	− (SARS)	NA
− OCS	5.3 (2.8)			2.1 (2.2)	
+ OCS	8.5 (6.4)			2.0 (2.0)	
Poyurovsky 2001, *n* = 68	− (AIMS)	− (BAS)	NA	NA	NA
− OCS	2.7 (2.9)	0.3 (0.7)			
+ OCS	3.3 (3.7)	0.4 (1.1)			
Poyurovsky 2007, *n* = 110	− (AIMS)	NA	NA	− (SARS)	NA
− OCS	0.1 (0.7)			10.0 (1.2)	
+ OCS	0.4 (1.3)			9.9 (1.2)	
Sevincok 2006 *n* = 57	− (AIMS)	− (BAS)	NA	− (SARS)	NA
− OCS	0.0	0.0		0.0	
+ OCS	0.0	0.0		0.0	
Tibbo 2000, *n* = 52	− (ESRS)	NA	− (ESRS)	+ (ESRS), *p* = 0.08	NA

*AIMS* Abnormal Involuntary Movement Scale, *BAS* Barnes Akathisia Scale, *HAS* Hillside Akathisia Scale, *SARS* Simpson-Angus Rating Scale, *CRS* Bush-Francis Catatonia Rating Scale, *ESRS* Extrapyramidal Symptom Rating Scale, *NA* not addressed

+ Significant association

− Non-significant association

The aim of the current study is to evaluate if and to what extent, the presence of OCS in patients and in their unaffected siblings is associated with the presence of more various motor symptoms (dystonia, akathisia, dyskinesia and parkinsonism). Siblings of patients with schizophrenia have a higher than average liability for psychosis. With regard to most signs and symptoms associated with schizophrenia, siblings have an in-between-position—less severe than patients, but more severe than controls—while, on the other hand, the siblings are free of confounding influences of the psychotic illness and anti-psychotic medication. The fact that motor symptoms are strongly associated with anti-psychotic medication—as well as OCS to a somewhat lesser extent—makes it interesting to evaluate whether findings in patients are also found in their healthy siblings. First, we will evaluate the cross-sectional relationship at baseline between OCS and motor symptoms in patients and their siblings. Second, we will examine whether the course of OCS is associated with a similar course of motor symptoms in patients and their siblings. We will divide the patients into four groups: a group with no OCS, with persistent OCS and two “changing” groups—with OCS at just one of the two assessments. Subsequently, we will determine whether motor symptoms change along with OCS. Third, we will seek to establish whether OCS at baseline predicts motor symptoms 3 years later and vice versa, in both patients and siblings.

To the best of our knowledge, no study has evaluated the course of motor symptoms and the relationship with OCS over time. Nor has the association between OCS and motor symptoms been assessed in healthy relatives of patients with schizophrenia. Identifying associations between motor symptoms and OCS in patients with schizophrenia, and providing longitudinal insight into the course of OCS and motor symptoms may have important treatment implications, such as the choice of antipsychotic medication. Furthermore, it adds information to the discussion concerning “the schizo-obsessive subtype”. Moreover, if comparable associations between OCS and motor symptoms are found in siblings and patients, this would support the hypothesis that an OCS–motor symptom association is at least partly independent of the confounding effect of medication. Since overlapping brain circuits are associated with OCS, motor symptoms and schizophrenia, the results might indirectly provide information about the brain regions involved.

## Methods

### Study design and participants

The study sample was part of the multicenter study ‘Genetic Risk and Outcome in Psychosis’ (GROUP), in which about 1000 patients and 1000 siblings were included. Baseline and 3-year follow-up assessments of patients and siblings with complete data on relevant outcome measures were included in the current study. The recruitment procedure and population characteristics have been described in detail elsewhere [21]. In short, inclusion criteria for patients and siblings were (1) age range of 16–50 years and (2) good command of the Dutch language. Patients had to meet DSM-IV-TR criteria for a non-affective psychotic disorder [4] which was assessed with the Comprehensive Assessment of Symptoms and History (CASH [3]) in three out of the four testing sites or the Schedules for Clinical Assessment for Neuropsychiatry version 2.1 (SCAN [48]) in one out of the four sites. An additional inclusion criterion for the sibling group was the absence of a lifetime psychotic disorder. All participants provided written informed consent prior to their inclusion in the study, which was approved by the accredited Medical Ethics Review Committee (METC).

### Clinical measures

Sociodemographic data on age, gender, education level, age of onset, duration of illness, and medical treatment were collected. Diagnosis according to DSM-IV was assessed. Current diagnosis of cannabis abuse and dependence was assessed using the Composite International Diagnostic Interview (CIDI [29]). Severity of positive and negative symptoms and general psychopathology in patients were evaluated with the Positive and Negative Syndrome Scale (PANSS [19]) according to the three-factor model. The Community Assessment of Psychic Experiences (CAPE [41]), a 42-item self-report questionnaire, was used to establish the frequency and associated distress with mild psychotic experiences in siblings.

A neuropsychological test-battery with seven cognitive tasks based on subtests of the Wechsler Adult Intelligence Scale-Third Edition (WAIS-III [46]) was administered, resulting in an IQ-score.

Severity of OCS was measured with the Yale-Brown Obsessive Compulsive Scale (YBOCS [16]), which has been validated for the assessment of OCS in schizophrenia [8, 11]. Based on interpretation guidelines, we defined the presence of clinically relevant OCS as a YBOCS total score of at least 8, representing mild symptom severity [16].

The motor section of the Unified Parkinson Disease Rating Scale (UPDRS) was used to assess parkinsonism [25]. A total score of at least 14 on the UPDRS and at least one item scored as mild symptoms was required to qualify as mild parkinsonism. The Abnormal Involuntary Movement Scale (AIMS [17]) was used to assess dyskinesia One item scoring at least mild ≥ 1 on the AIMS was required to qualify as mild dyskinesia. Akathisia was assessed using the Barnes Akathisia Rating Scale (BARS) [6]. To qualify as mild akathisia, the BARS global score, a composite overall severity score requiring both subjective and objective symptoms to be present, needed to be at least mild (≥ 2). One item was added to the BARS to measure acute dystonia with a severity score from 0 to 4. For the BARS dystonia scale again a mild symptom score, ≥ 1, was required to qualify as dystonia. A composite motor score was generated, defined as having at least one motor symptom scored as “mild” on any of the mentioned instruments assessing motor symptoms. To obtain enough power, we lowered the threshold for siblings on the Y-BOCS and the motor symptom scales to any score.

Before the start of the study all interviewers participated in extensive training workshops to practice the assessments of all measures used in the GROUP project (for detail see [21]).

### Statistical analysis

Statistical analyses were performed using the Statistical package for Social Sciences (SPSS version 23.0, Chicago, IL, US). Pearson Chi-Square tests, *T* test and logistic regression were used when appropriate, to determine demographic, illness characteristics and prevalences. Estimates were adjusted for potential confounders (gender, age, ethnicity, marital status, education level, IQ, and in patients—GAF-scores, clozapine and first-generation anti-psychotic use, anti-depressant use, duration of illness and PANSS scores; and in siblings—CAPE scores). Because of potential overlap, the PANSS-general scale score was not entered as a confounder. Confounding variables were entered successively in the logistic regression with motor symptoms as the independent variable and the OCS status as the dependent variable. The potential confounders were included in the analyses if they had a meaningful impact on the effect estimate (defined as more than 5%) or if the model fit significantly improved (− 2 log likelihood method), and were included in the final models. Confounders were selected for patients and siblings and the two assessment periods, separately. In patients and siblings, sociodemographic and clinical characteristics were compared between subjects with OCS and without OCS using Pearson Chi-square tests and *T* tests. The cross-sectional association between OCS and motor symptoms was assessed using logistic regression, with and without correction for confounders. For the longitudinal pairwise comparison between OCS change-groups and motor symptoms in patients, we used the Pearson Chi-square test and logistic regression to assess prevalences and odds ratios, again with and without correction for confounders. The cross-assessment cross-symptom associations in patients and siblings were evaluated using logistic regression.

## Results

### Sociodemographic and clinical characteristics in patients

Data were present on both OCS and motor symptoms at both baseline assessment and 3-year follow-up for 726 patients. At least one motor symptom was scored as mild during at least one assessment in 344 patients (47.4%), while 182 patients (25.1%) had OCS in at least one assessment. OCS at baseline were present in 106 patients and OCS at follow-up were present in 117 patients. Of these 41 had OCS at both assessments. At baseline 252 patients had motor symptoms and 210 at follow-up, of whom 102 had persistent motor symptoms.

Table 2 presents the socio-demographic and clinical characteristics of patients with and without OCS at baseline. Patients with OCS more often used anti-depressants, more often used clozapine, had lower GAF-scores and higher scores on all three PANSS scales (Table 2).

**Table 2:** Differences in patient characteristics at baseline and follow-up associated with OCS status

Patient *n* = 726	Baseline			Follow-up		
	OCS −, *n* = 620 (85.4%)	OCS +, *n* = 106 (14.6%)		OCS −, *n* = 609 (83.9%)	OCS +, *n* = 117 (16.1%)	
Gender (male)	468 (75.5%)	84 (79.2%)	*χ*^2^ = 0.7 (1), *p* = 0.402	461 (75.7%)	91 (77.8%)	*χ*^2^ = 0.23 (1), *p* = 0.629
Age (mean, sd)	27.5 (7.4)	26.4 (6.7)	(*t* = 1.5, *p* = 0.145)	30.8 (7.6)	30.0 (5.9)	(*t* = 1.0, *p* = 0.321)
Duration of illness (mean, sd)	4.3 (4.2)	5.0 (3.5)	(*t* = − 1.5, *p* = 0.127)			
Education level			*χ*^2^ = 3.0 (3), *p* = 0.386			
Primary school or missing	68 (11.0%)	15 (14.1%)				
Secondary basic	376 (60.6%)	69 (65.1%)				
Secondary advanced/college	176 (28.4%)	22 (20.8%)				
Ethnicity (Caucasian)	511 (82.4%)	84 (79.2%)	*χ*^2^ = 0.6 (1), *p* = 0.532			
Marital status (with partner)	63 (10.2%)	5 (4.7%)	*χ*^2^ = 3.2 (2), *p* = 0.206			
Clozapine use	93 (16.2%)	27 (27.8%)	*χ*^2^ = 7.6 (1), *p* = 0.006**	76 (14.7%)	31 (28.4%)	*χ*^2^ = 11.9(1), *p* = 0.001**
Classic antipsychotic use	153 (26.6%)	24 (24.7%)	*χ*^2^ = 0.2 (1), *p* = 0.686	67 (13%)	16 (14.7%)	*χ*^2^ = 0.2(1), *p* = 0.636
Antidepressant use	79 (13.9%)	22 (22.2%)	*χ*^2^ = 4.5 (1), *p* = 0.03*	No information		
Cannabis use	118 (19.0%)	18 (17.0%)	*χ*^2^ = 0.3 (1), *p* = 0.617	51 (8.4%)	9 (7.7%)	*χ*^2^ = 0.06(1), *p* = 0.806
IQ (mean, sd)	96.5 (16.2)	95.9 (15.3)	(*t* = 0.4, *p* = 0.709)	99.3 (16.8)	96.9 (16.6)	(*t* = 1.3, *p* = 0.212)
GAF (mean, sd)	57.8 (15.7)	51.8 (16.9)	(*t* = 3.4, *p* = 0.001)**	62.5 (15.7)	52.1 (14.7)	(*t* = 6.3, *p* < 0.0005)**
PANSS positive scale (mean, sd)	11.8 (5.0)	14.4 (5.5)	(*t* = −4.9, *p* < 0.0005)**	10.3 (4.2)	13.5 (4.7)	(*t* = −7.4, *p* < 0.0005)**
PANSS negative scale (mean, sd)	13.2 (5.7)	14.5 (5.6)	(*t* = − 2.1, *p* < 0.035)*	11.5 (5.0)	12.7 (4.8)	(*t* = −2.5, *p* < 0.013)*
PANSS general scale (mean, sd)	26.2 (7.7)	31.2 (8.2)	(*t* = −6.0, *p* < 0.001)**	23.0 (6.7)	28.0 (7.4)	(*t* = −7.3, *p* < 0.0005)**
Composite motor scale	203 (32.7%)	39 (36.8%)	*χ*^2^ = 0.67 (1), *p* = 0.414	163 (26.7%)	47 (40.2%)	*χ*^2^ = 8.60 (1), *p* = 0.003**
Akathasia	49 (7.9%)	13 (21.0%)	*χ*^2^ = 2.2 (1), *p* = 0.14	37 (6.1%)	16 (13.7%)	*χ*^2^ = 8.42 (1), *p* = 0.004**
Dystonia	21 (3.4%)	5 (4.7%)	χ^2^ = 0.5 (1), *p* = 0.691	20 (3.3%)	6 (5.1%)	*χ*^2^ = 0.97 (1), *p* = 0.326
Dyskinesia	125 (20.2%)	23 (21.7%)	χ^2^ = 0.1(1), p = 0.717	92 (15.1%)	25 (21.4%)	*χ*^2^ = 2.85 (1), *p* = 0.09
Parkinsonism	67 (10.8%)	11 (10.4%)	*χ*^2^ = 0.0(1), *p* = 0.895	70 (11.5%)	21 (17.9%)	*χ*^2^ = 3.73 (1), *p* = 0.053

*p ≤ 0.05

**p ≤ 0.01

### Sociodemographic and clinical characteristics in unaffected siblings

Data were present on both OCS and motor symptoms at both the baseline assessment and the 3-year follow-up for 761 siblings. Of the unaffected siblings, 98 (14.2%) had OCS during at least one assessment. Any score on a motor symptom scale was present in 350 (46.0%) siblings for at least one assessment. At baseline 55 siblings had OCS and 67 had OCS at follow-up. Of these 14 had OCS at both assessments. At baseline 232 siblings had motor symptoms and 244 at follow-up, of whom 126 had persistent motor symptoms.

Table 3 presents the socio-demographic and clinical characteristics of siblings with and without OCS at baseline. Siblings reporting OCS at baseline showed higher CAPE scores, but did not differ significantly on other demographic or clinical characteristics (Table 3).

**Table 3:** Differences in sibling characteristics at baseline associated with OCS status

Sibling characteristics at baseline (*n* = 761)	OCS −, *n* = 706 (92.8%)	OCS +, *n* = 55 (7.2%)	
Gender (male)	317 (44.9%)	17 (30.9%)	*χ*^2^ = 4.1 (1), *p* = 0.044*
Age (mean, sd)	27.6 (8.2)	28.1 (5.9)	*t* = − 0.4, *p* = 0.671
Education level			*χ*^2^ = 6.3 (3), *p* = 0.100
Primary school or missing	48 (6.8%)	3 (5.5%)	
Secondary (VMBO HAVO MBO)	365 (51.7%)	20 (36.4%)	
Secondary advanced level or college	293 (41.5%)	32 (58.2%)	
Ethnicity (Caucasian)	616 (87.3%)	48 (87.3%)	*χ*^2^ = 0.0 (1), *p* = 0.996
Marital status (with partner)	277 (39.2%)	23 (41.8%)	*χ*^2^ = 1.5 (1), *p* = 0.457
Cannabis use	33 (4.7%)	1 (1.8%)	*χ*^2^ = 1.0 (1), *p* = 0.323
IQ (mean, sd)	104.3 (15.7)	103.9 (15.4)	*t* = 0.2, *p* = 0.864
CAPE positive (mean, sd)	0.19 (0.18)	0.25 (0.25)	*t* = − 2.4, *p* = 0.015*
CAPE negative (mean, sd)	0.54 (0.37)	0.75 (0.49)	*t* = − 3.8, *p* = 0.000**
CAPE depressive (mean, sd)	0.62 (0.38)	0.81 (0.50)	*t* = − 3.8, *p* = 0.001**
Composite scale any motor sympt	214 (30.3%)	18 (32.7%)	*χ*^2^ = 0.1(1), *p* = 0.708
Akathisia	22 (3.1%)	6 (10.9%)	*χ*^2^ = 8.7(1), *p* = 0.003**
Dystonia	5 (0.7%)	0(0%)	*χ*^2^ = 0.4(1), *p* = 0.531
Dyskinesia	60 (8.5%)	5 (9.1%)	*χ*^2^ = 0.0(1), *p* = 0.880
Parkinsonism	180 (25.5%)	15 (27.3%)	*χ*^2^ = 0.1 (1), *p* = 0.771

### Cross-sectional association between OCS and motor symptoms at baseline and 3-year follow-up in patients

To assess the association between OCS and motor symptoms, we used logistic regression analyses calculating odds ratios. The following potential confounders were entered in the logistic regression: gender, age, ethnicity, marital status, education level, IQ, GAF-scores, clozapine and first-generation anti-psychotic use, anti-depressant use, duration of illness and PANSS scores. For the baseline assessment, only GAF-score, clozapine use and the PANSS-negative score were identified as confounders, using the composite motor scale as independent variable and OCS status as outcome.

At baseline, the presence of OCS showed no significant association with any of the motor scales assessed. This result remained unchanged after adjusting for the identified confounders (Table 4).

**Table 4:** Results of naïve logistic regression analyses, comparing mild motor symptoms in patients and siblings without OCS with patients with OCS at baseline and follow-up

	Unadjusted model T0	Fully adjusted model T0	Unadjusted model T1	Fully adjusted model T1
	OR (95% CI)	OR (95% CI)	OR (95% CI)	OR (95% CI)
Patients				
Composite scale	1.2 (0.8–1.8)	0.7 (0.6–1.5)^a^	1.8 (1.2–2.8)**	1.3 (0.8–2.1)^b^
Mild akathisia	1.6 (0.8–3.1)	1.3 (0.6–2.8)^a^	2.4 (1.3–4.6)**	2.2 (1.1–4.4)^b,^*
Mild dystonia	1.4 (0.5–3.8)	1.5 (0.5–4.2)^a^	1.6 (0.6–4.1)	0.7 (0.2–2.6)^b^
Mild parkinsonism	1.0 (0.5–1.9)	0.8 (0.4–1.7)^a^	1.7 (1.0–2.9)	0.9 (0.4–1.8)^b^
Mild dyskinesia	1.1 (0.7–1.8)	0.9 (0.5–1.6)^a^	1.5 (0.9–2.5)	1.4 (0.8–2.5)^b^
Siblings				
Composite scale	1.1 (0.6–2.0)	1.2 (0.7–2.2)^c^	1.0 (0.6–1.8)	1.0 (0.6–1.7)^d^
Akathisia	3.8 (1.5–9.8)**	3.8 (1.5–9.9)^c,^**	1.6 (0.5–5.5)	1.5 (0.4–5.3)^d^
Parkinsonism	1.1 (0.6–2.0)	1.2 (0.6–2.2)^c^	1.2 (0.7–2.1)	1.2 (0.7–2.0)^d^
Dyskinesia	1.0 (0.4–1.8)	1.2 (0.4–3.0)^c^	1.2 (0.5–2.9)	1.2 (0.5–2.8)^d^

^a^Included confounders patients T0: GAF score, clozapine use, PANSS-negative

^b^Included confounders patients T1: GAF score, IQ, PANSS-negative

^c^Included confounders siblings T0: gender

^d^Included confounders siblings T1: marital status

*p ≤ 0.05

**p ≤ 0.01

For the follow-up assessment, GAF-score, IQ and PANSS-negative score were identified as confounders. The PANSS-positive was entered as a potential confounder for both assessments, but did not independently contribute to the model.

At follow-up, OCS was positively associated with the composite motor scale and akathisia; presence of OCS raised the odds for suffering from motor symptoms by 1.8 (CI 1.2–2.8, *p* = 0.004), and from akathisia by 2.4, (CI 1.3–4.5, *p* = 0.005), respectively. The other motor symptoms were not significantly associated with the presence of OCS. After adjusting for the identified confounders only the association between OCS and akathisia remained significant (OR 2.2, CI 1.1–4.4, *p* = 0.033) (Table 4).

### Cross-sectional association between OCS and motor symptoms at baseline and 3-year follow-up in unaffected siblings

Note that OCS and motor symptom cut-offs for siblings are lower than for patients, which makes direct comparison of reported percentages impossible. In siblings at baseline, the presence of OCS increased the odds of suffering from akathisia by 3.8 (CI 1.5–9.8), which remained unchanged after adjusting for the identified confounder gender. The other potential confounders, age, ethnicity, marital status, education level, IQ and CAPE scores did not have a significant effect on the outcome. The other motor symptoms showed no significant association with OCS (Table 4).

At the follow-up assessment, marital status was identified as a confounder and OCS was no longer associated with any motor symptom (Table 4). The sibling sample consisted of significantly more females, while the patient sample contained more males. No sibling had a dyskinesia score at any assessment.

### Longitudinal pairwise comparison between OCS change-groups and motor symptoms in patients

**Fig. 1:**
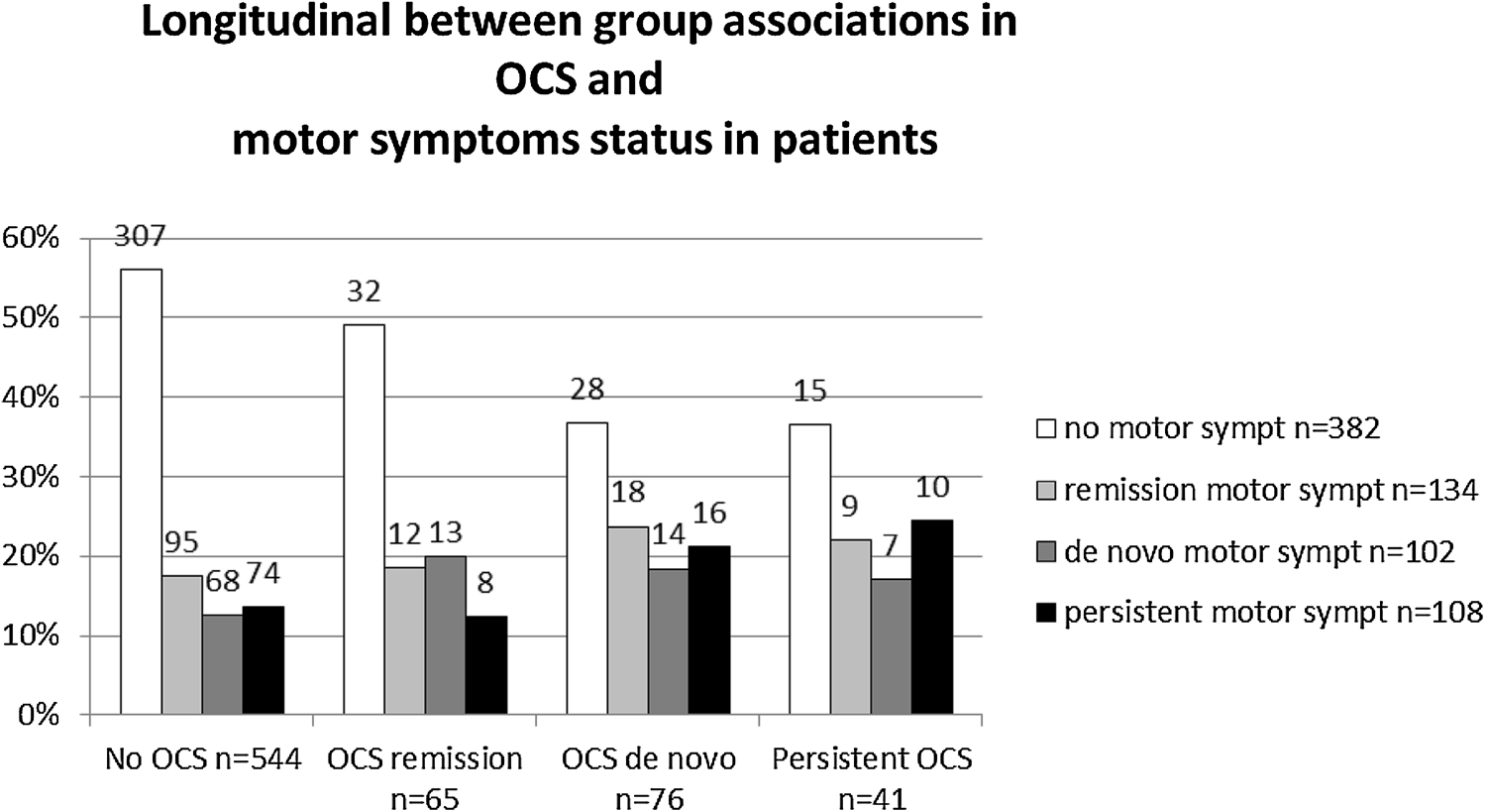
Distribution of motor symptom status per OCS status group expressed in percentage of the OCS group total in patients. Information on number of patients per group are added

To study the course of OCS and its relationship with the course of motor symptoms both OCS and motor symptoms were categorized into four groups as was done in our previous analyses [37, 38]: (1) a no symptom group, (2) a remission group, showing symptoms at baseline assessment only, (3) a de novo group, showing symptoms at follow-up, but not at baseline and (4) a persistent group, showing symptoms at both assessments. Subsequently, the relationship between those groups and motor symptoms was analyzed (Fig. 1). In patients, the association between the four OCS-groups and the four motor symptom-groups was significant (*χ*^2^ = 18.1 (9), *p* = 0.034).

Subsequent pair-wise comparisons regarding the four OCS-groups revealed that the OCS de novo group significantly differed compared to the no-OCS group (*χ*^2^ = 10.5 (3), *p* = 0.015). The persistent OCS group is very similar to the OCS de novo group in motor symptom prevalences, but analyses did not reach significance, probably because of the group size being almost half the size of the OCS de novo group [*χ*^2^ = 6.9 (3), *p* = 0.076]. All other pairwise comparisons failed to show a significant association.

To evaluate the effect of the OCS groups on motor symptoms at baseline and follow-up, we subsequently performed exploratory analyses: a logistical regression focusing on those groups and symptoms showing significant results in previous analyses. We performed a pair-wise comparison between the no-OCS group and the de novo group and the no-OCS group and the persistent OCS group, looking at the composite motor scale and akathisia at both assessments. Table 5 shows the results. Akathisia is associated with both the de novo OCS group and the persistent OCS group at both assessments; for the persistent group, the association loses significance at baseline after correcting for confounders. The composite scale is also associated with the de novo and persistent group, but this association loses significance after correction for confounders.

**Table 5:** No-OCS group compared to the OCS de novo group and the persistent OCS group regarding their composite motor score and akathisia at baseline and follow-up

Patients, No-OCS = reference group, Unadjusted and adjusted	OCS de novo, OR (95%-CI)		Persistent OCS, OR (95%-CI)	
Composite scale T0	1.8 (1.1–2.9)*	1.5 (0.9–2.5)^a^	1.9 (1.0–3.6)*	1.6 (0.8–3.2)^a^
Mild akathisia T0	2.6 (1.3–5.2)**	2.2 (1.0–4.8)^a,^*	2.3 (0.9–5.9)	2.0 (0.7–5.6)^a^
Composite scale T1	1.8 (1.1–3.0)*	1.7 (1.0–3.0)^b^	2.0 (1.0–3.8)*	1.8 (0.9–3.6)^b^
Mild akathisia T1	2.7 (1.3–5.8)*	2.6 (1.4–6.1)^b,^*	3.0 (1.2–7.8)*	2.3 (0.8–6.7)^b^

^a^Corrected for confounders baseline: clozapine, GAF score and PANSS negative

^b^Corrected for confounders follow-up: PANSS negative, GAF, IQ

*p < 0.05; **p < 0.01

### Longitudinal association between OCS and motor symptoms in siblings

**Fig. 2:**
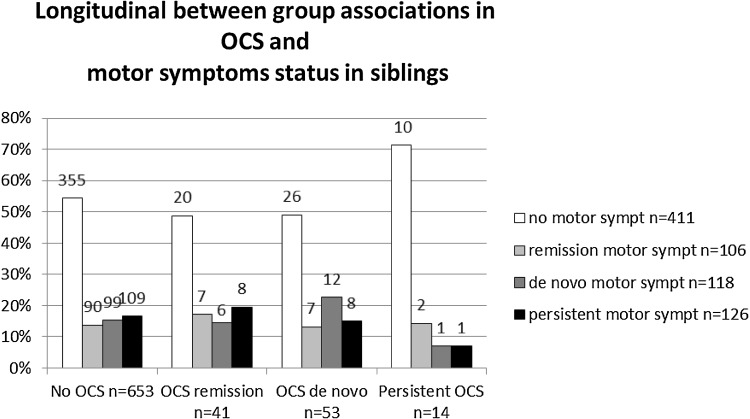
Distribution of motor symptom status per OCS status group expressed in percentage of the OCS group total in siblings. Information on number of siblings per group are added

Siblings were grouped according to their OCS or motor symptom status (none, remission, de novo or persistent). No significant association between OCS and motor symptom status were found (Fig. 2). Note that the number of siblings scoring on OCS particularly is small, resulting in some very small cell counts. Motor symptoms tend to go into remission in about 50% of all cases, independent of the OCS status of siblings. In siblings, persistent OCS is very rare: only 14 siblings (1.8%) had OCS at both assessments. Having OCS at one assessment is more common, 5.4% at baseline and 7.0% at follow-up.

### Cross-assessment cross-symptom associations in patients and siblings

In patients, the presence of motor symptoms at baseline is associated with OCS at follow-up, (OR 1.8, CI 1.2–2.8, *p* = 0.003). Conversely, OCS at baseline is not significantly associated with motor symptoms at follow-up (OR 1.5, CI 0.9–2.2, *p* = 0.090).

In siblings, motor symptoms at baseline are not significantly associated with OCS at follow-up (OR 0.8, CI 0.5–1.4, *p* = 0.501), nor is OCS at baseline associated with motor symptoms at follow-up (OR 0.9, CI 0.5–1.6, *p* = 0.860).

## Discussion

The main findings of our study are as follows. In patients, we found no cross-sectional association between OCS and any motor symptoms at baseline, but we did find a cross-sectional association between OCS and akathisia and the composite motor score at follow-up. The association between akathisia and OCS remained significant after correcting for confounders. In siblings the only significant association was again found between the presence of OCS and akathisia, however, this association was only present at baseline. When divided into four groups, the persistent OCS group and the OCS de novo group were associated with more akathisia.

Compared to the results of previous studies (Table 1), it is remarkable that we find an association only with akathisia. Of the five studies assessing akathisia, only one found a significant association. The associations between OCS and parkinsonism found in four out of seven previous studies and between OCS and dyskinesia found in four out of eight studies, on the other hand, were not replicated in the current study.

The following factors may explain the differences between our results and those of earlier studies.

Comparing AIMS scores for dyskinesia, which were provided in seven studies we see considerable heterogeneity in scores, between zero and 8.5 (Table 1), indicating substantial differences between the samples. In our sample at baseline, the mean AIMS-score was 0.5 (SD 1.3) in patients without OCS and 0.4 (SD 1.0) in patients with co-morbid OCS. This is a relatively low score compared to most other studies, indicating potential fundamental differences between our study sample and other study samples. The GROUP sample with a mean age of 27 years and a 4-year duration of illness is relatively young compared to samples from earlier studies, in which the mean age ranged from 30 to 45 years and the duration of illness ranged from 9 to 22 years [28, 32, 34]. Particularly dyskinesia and to a lesser extent parkinsonism may develop over a period of years; therefore, the illness duration of our sample might be too short to have allowed the development of those motor symptoms. Another explanation for the differences with former studies may be that anti-psychotic prescription protocols have changed in the past 15 years. Modern anti-psychotics have become an alternative to classical anti-psychotics, in particular when motor side-effects are present, and new insights found that first-generation anti-psychotics are best prescribed in much lower dosage than had been done previously. In the GROUP sample, relatively few patients (about 25%) used first-generation anti-psychotics, and dosages were relatively low. Both the prescription of lower dosages and the option of alternative medication with fewer motor symptom side effects will have reduced motor symptoms over the course of time, resulting in lower motor symptom prevalences in the more recent studies.

Our finding that OCS is associated with akathisia, which has not been found in previous studies, might be explained by our large sample size with enough power to find genuine differences. In a previous study, the unexpected emergence of akathisia was reported in 40% of SSRI-treated OCD patients after they were given one challenge with amisulpride (400 mg). In OCD patients the serotonergic transporter binding site is reduced compared to healthy volunteers [35], while a 20% reduction of dopamine receptor availability in the left caudate has been reported [13]. The modern anti-psychotics not only block the dopamine receptors but also interact with the different serotonin receptors [15]. Since OCD patients have deficiencies in both receptor systems, a comparable disturbance to those systems may underlie the vulnerability of schizophrenia patients with comorbid OCS for motor side-effects and potentially motor symptoms in general. Furthermore, in a review on drug-induced akathisia by Stahlet al. [40], akathisia is linked to a decrease in dopaminergic neurotransmission, which through a compensatory enhancement of adrenergic projections, results in a mismatch of activity between the shell and the nucleus of the nucleus accumbens. Although OCD is associated with the whole cortico-striato-thalamic-cortical circuitry [12, 31], Figee et al. found specific differences in the nucleus accumbens activity between OCD patients and healthy volunteers [14]. Therefore, a malfunctioning of the nucleus accumbens could possibly explain the co-occurrence between OCS and motor symptoms we find in our study in both patients and their siblings.

An alternative explanation might be that overlap between OCS and akathisia results in false positive cases. Specifically, the subjective feeling of restlessness, part of the akathisia, is close to the distress seen in patients with the urge to control or suppress compulsions in response to intrusive thoughts [5]. Little is known about the early course of OCD and even less is known concerning the early course of OCD in schizophrenia, but a retrospective study reported that “anxiety” and “lacking self-trust” were frequent first signs of developing OCD [18]. Specifically, the anxiety could partially overlap with mild akathisia. This could explain why the de novo-group has relatively high akathisia scores at baseline, without the OCS being present yet. The fact, though, that we find no consistent covariation between OCS and motor symptoms renders an overlap in symptoms unlikely as full explanation for their association.

Longitudinal analyses showed that the difference in association with OCS and motor symptoms in patients, absent at baseline but present at follow-up, were mainly driven by significantly higher akathisia and composite motor scores in the group that developed OCS de novo and in the persistent group (Table 5). The strength of the association at follow-up measured in odds ratios, between OCS and akathisia was similar for the persistent group and the de novo group. Unlike the de novo and persistent group, the OCS remission group did not differ significantly at any assessment regarding its motor symptoms, compared to the no-OCS group. In patients, motor symptoms at baseline were associated with OCS at follow-up, but not the other way around.

The longitudinal results partly stand in line with results of previous analyses from the GROUP data, comparing OCS status groups and the course of PANSS and GAF scores [38] as well as cognitive functioning over the 3 year assessment period [37]. In all three studies at baseline, the group which was going to develop OCS at follow-up—the OCS de novo group—already showed more impairment than the no-OCS group. In the current study, at baseline, the OCS de novo group already had more motor symptoms and these symptoms persisted over time and remained more or less stable. Similar to the motor symptoms, patients in the OCS de novo group reported stable higher psychopathology on the PANSS and GAF compared to the no-OCS group. On cognitive performance, the OCS de novo group had deteriorated on the set-shifting task at follow-up, while the OCS remission group remained stable or improved on all tasks [25, 26]. This is in line with the cross-trait cross-assessment result, showing that in patients motor symptoms at baseline were associated with OCS occurrence at follow-up, but not the other way around. Taken together, our results might indicate that motor symptoms can appear before the emergence of OCS along with higher PANSS scores, poorer overall functioning and poorer cognitive functioning in certain tasks.

In unaffected siblings, we also found an association between akathisia and OCS, but not with the other motor symptoms. It is remarkable that of the five motor symptoms assessed, in both patients and siblings, only akathisia showed a positive association with OCS, which may indicate a link between akathisia and OCS in psychotic and psychosis-prone individuals, which could be explained by malfunction of neurobiological areas causing both OCS and akathisia. For instance, disturbances in the functioning of the nucleus accumbens may underlie both OCS and akathisia.

In siblings, this association is present at baseline, but disappears at follow-up. At baseline, six out of 28 siblings with akathisia have OCS. At follow-up, three out of 23 akathisia patients had OCS. This small difference of three siblings accounts for the presence or absence of a significant association, which makes it difficult to draw conclusions. Although 761 siblings were included and assessed twice, the proportion of siblings with OCS or motor symptoms was small. In lowering the threshold to any score on both the motor symptom scale and the Y-BOCS, we have probably included false positive cases.

Several limitations should be considered in interpreting the results of our study. First, the fact that there were only two assessments over a 3-year period is an important limitation, because many events with substantial influences on the course of illness may have taken place unnoticed in between assessments.

Second, although we tried to correct for possible confounders, it is unlikely that we were able to correct for all influencing variables, while it is possible that we corrected for partly overlapping variables. Information on antipsychotic dosage and on anti-cholinergic agents is missing, so we were unable to correct for these potential confounders. Third, we decided not to correct for multiple comparisons, because we compared the three groups on four strongly related items and the results are very consistent. Notwithstanding this, there is a chance that our results are false positive. Therefore replication of these results is important before strong conclusions can be drawn.

Fourth, the mean severity of OCS and motor symptoms is relatively low, especially in the sibling sample. Despite these limitations, our current study has many methodological strengths compared to former studies. Few studies assessed the association between co-occurring OCS and motor symptoms in patients suffering from schizophrenia. Results have been inconsistent, numbers of patients included were small and in most studies, only one or two motor symptoms were assessed, while no correction for possible confounders was made. Until now the longitudinal course and interaction of OCS and motor symptoms have not been studied nor has this been examined in unaffected siblings. Our sample is by far the largest ever to have evaluated motor symptoms in patients with schizophrenia, with and without OCS. Because of its multi-center design including patients from different areas and clinical settings in contrast to the previous studies, our results are more applicable to the general patient population.

In conclusion, our results show that few motor symptoms are associated with OCS in schizophrenia, but suggest that the presence of persistent OCS and OCS de novo are associated with akathisia during the course of the illness. Both at baseline and follow-up, patients with persistent OCS and patients developing OCS de novo have more combined motor symptoms and akathisia compared to patients without OCS. Moreover, motor symptoms may predict OCS at follow-up. In unaffected siblings, we found an association between akathisia and OCS at baseline as well, which is lost at follow-up. However, although the sibling sample is relatively large, severity of OCS or motor symptoms in siblings is low, therefore, this finding is not robust.

Our results have the following implications: Clinicians should be alert to the possibility that patients with comorbid OCS might have a higher risk of developing akathisia, especially since akathisia is difficult to diagnose and can be easily misinterpreted as psychosis-related agitation [9]. The fact that the co-occurrence of akathisia and OCS is also found in siblings implies that akathisia is at least partly caused by factors other than antipsychotic medication. In this respect, disturbances of function of the nucleus accumbens is of interest. In the future more longitudinal studies with smaller intervals would help, if our results are replicated, in identifying a pattern of emergence of symptoms, some of which might have a certain “predictive” value. Finally, patients would benefit from treatment trials, identifying optimal medication protocols for the treatment of comorbid OCS in schizophrenia, monitoring side-effects, such as akathisia.
